# Position and Dimensions of the Mandibular Condyle in Various Anterior–Posterior Skeletal Patterns: A CBCT Imaging Study in a Sample of Iranian People

**DOI:** 10.1155/ijod/5895594

**Published:** 2024-12-27

**Authors:** Zahra Vasegh, Yaser Safi, Kazem Dalaei, Mehdi Hosseinzadeh, Nasim Tayari

**Affiliations:** ^1^Department of Oral and Maxillofacial Radiology, School of Dentistry, Shahid Beheshti University of Medical Sciences, Tehran, Iran; ^2^Department of Orthodontics, School of Dentistry, Shahid Beheshti University of Medical Sciences, Tehran, Iran; ^3^School of Dentistry, Shahid Beheshti University of Medical Sciences, Tehran, Iran

**Keywords:** cone beam computed tomography, mandibular condyle, temporomandibular joint

## Abstract

**Purpose:** The aim of this comparative observational study is to evaluate and compare the size and position of the condyle among male and female patients with different skeletal patterns in the anterior-posterior dimension using cone beam computed tomography (CBCT) images.

**Materials and Methods:** CBCT images of 120 patients, all prepared for other treatment purposes under the same conditions, were included in the study. The patients were classified into three groups—class I, class II, and class III—based on ANB angles and Wits analysis. The size of the condyle was measured in terms of width, height, and length. The position of the condyle was assessed by measuring the superior joint space (SS), anterior joint space (AS), and posterior joint space (PS) on the right and left sides separately. The measurements and results were analyzed using analysis of covariance (ANCOVA) and Bonferroni analysis. A statistical significance level of *p*  < 0.05 was considered.

**Results:** The study found no statistically significant differences in the size of the SS and AS (*p* = 0.481 and *p* = 0.392, respectively) across different skeletal patterns. However, the size of the PS was significantly greater in class I subjects compared to class III subjects (*p* = 0.015). There were no statistically significant differences in condyle height and width among the different skeletal patterns (*p* = 0.367 and *p* = 0.720, respectively). In contrast, condyle length was statistically significant in class II individuals (*p* = 0.002) and was the lowest among the other skeletal pattern groups.

**Conclusions:** Based on the results obtained, class I individuals have lower PS values compared to class III individuals. Additionally, class II individuals have shorter condyle lengths compared to those in class III and class I.

## 1. Introduction

The temporomandibular joint (TMJ) is among the most complex joints in the human body and serves as a crucial growth site within the craniofacial complex due to its unique anatomical, histological, and biomechanical properties. Its cartilaginous tissue is capable of remodeling in response to external forces, even after growth has ceased. Therefore, the condyles are not only primary targets in orofacial orthopedics, but also key determinants of long-term stability in orthognathic surgeries. TMJ morphology can vary significantly among individuals and may even differ between the right and left sides of the same individual. Given the close relationship between form and function, orthodontists often anticipate a correlation between condylar characteristics and craniofacial morphology [1, 2]. The morphology and position of the TMJ are critical for the stability of orthodontic treatments and can be influenced by several factors, including age, gender, growth patterns, muscular activity, and occlusal changes [3]. The type of skeletal pattern can impact the morphology and position of the condyle and the glenoid fossa [4]. The ANB angle is a recognized cephalometric measurement used to evaluate the anteroposterior relationship between the maxilla and mandible. Clinically, this angle is crucial for diagnosing skeletal relationships. Its measurement provides insight into whether a patient has a Class I, Class II, or Class III skeletal pattern, which in turn influences the therapeutic approach in both orthodontics and maxillofacial surgery. The ANB angle is a key factor in clinical treatment decision-making. Some studies found significant correlations between ANB and masticatory function, including masseter muscle activity and abnormal chewing patterns. These results highlight the ANB angle's role in determining treatment type, helping clinicians decide between camouflage orthodontics and surgical intervention [5]. Some studies have explored the correlation between serological variables and temporomandibular disorders (TMDs) in patients with systemic and chronic autoimmune diseases, such as rheumatoid arthritis (RA), which involve inflammation of the synovial joints. Certain serological factors may serve as predictive markers for specific TMDs [6].

Imaging plays a crucial role in accurate diagnosis. Conventional radiographic techniques used in orthodontic treatments, such as cephalometry and panoramic radiography, do not provide a clear, multidirectional view of the TMJ [7]. Cone beam computed tomography (CBCT) has proven valuable in detecting significant changes in condyle position in patients with TMJ disorders [8]. CBCT allows for the assessment of TMJ anatomy without the superimposition of structures, thereby, facilitating the analysis of bone structure, joint space, and dynamic function in three dimensions. This technology surpasses the limitations of other imaging techniques like panoramic radiography and traditional computed tomography [9, 10].

Various studies have examined the relationship between skeletal malocclusions and TMJ morphology, highlighting significant differences across Angle's classifications. For instance, some studies found that patients with Class III malocclusion exhibited significant changes in joint space, with a more anteriorly displaced condyle compared to those with Class I or II malocclusions [1]. Differences in condylar morphology, joint space, and fossa dimensions between skeletal patterns, suggesting that these variations could play a role in the development of TMDs across different skeletal classes. In particular, class III subjects were characterized by a wider mandibular fossa and a more superior condylar position, whereas Class II patients showed more pronounced changes in joint space and condylar height [11]. The morphology and position of the condyles are significant in orthodontics as they can influence the outcomes and stability of orthodontic treatments [1, 12]. The ideal position of the condyle within the glenoid fossa remains a fundamental question in dentistry [13, 14]. There is ongoing debate regarding the clinical importance of condylar positioning in the TMJ. The position of the condyle is the result of various dynamic processes, including growth, remodeling, and responses to functional and occlusal changes [15–17]. The impact of occlusion on the condyle–fossa relationship continues to be a subject of research. Some studies have recognized the role of occlusion in the relationship between the condylar process and the mandibular fossa [18–20]. Beyond the position, occlusion may also be associated with the vertical height, anteroposterior, and mediolateral thickness of the condylar head [13, 21]. The condyle–fossa relationship may or may not be associated with variables such as sex, age, and laterality (left and right sides), as suggested by various studies [22].

Orthognathic surgeries and growth modification treatments can affect the position of the condyle and the dimensions of the joint spaces. Numerous studies have indicated that orthognathic surgeries can alter the position of the condyle within the joint space, though these changes are often not significant in the long term [23, 24]. However, other studies have reported that such changes can be significant even over the long term [25, 26]. Changes in condylar position and joint spaces can impact the recurrence of surgical outcomes [27–29]. Growth modification treatments can also alter the position of the condyle, potentially causing its displacement [30]. Furthermore, the correction of Angle's Class I malocclusions using fixed orthodontic appliances has been associated with significant posterior movement of the condyle. The posttreatment position of the condyle is crucial as it can aid orthodontists in predicting the condylar position during treatment planning [31].

Significant variations in the dimensions and spatial orientation of the mandibular condyles within the glenoid fossae have been observed between male and female subjects with different sagittal skeletal relationships. These morphological differences, as demonstrated through CBCT analysis, are likely to correlate with specific malocclusion classifications. Consequently, these anatomical variations may influence the efficacy and long-term stability of orthodontic interventions. Therefore, understanding the extent of joint spaces and the condylar position across different anterior–posterior skeletal patterns is essential for optimizing outcomes in orthognathic surgery and growth modification treatments, aiding orthodontists and surgeons in treatment planning. Additionally, there is still a gap in comprehensive knowledge regarding the relationship between TMJ morphology and various types of malocclusions, particularly within the Iranian population. The aim of this study is to evaluate and compare the size and position of the right and left condyles in the glenoid fossa among male and female patients with different skeletal patterns in the anterior-posterior dimension using CBCT images.

## 2. Materials and Methods

This study was approved by the Ethics Committee of Shahid Beheshti University of Dental Medicine under the approval number IR.SBMU.DRC.REC.1401.021. Due to the retrospective nature of the study and the anonymization of patients' data in the final report, specific ethical considerations were not applicable.

From March 2019 to April 2023, a total of 153 CBCT images were collected from two oral and maxillofacial centers. The inclusion criteria required appropriate image quality, coverage of the examined area, symmetrical skeletal appearance, complete dentition (with or without the third molars), and participants aged over 16 years. Exclusion criteria included evidence of trauma or surgery, crossbite or open bite, a history of orthodontic or orthopedic treatment, congenital syndromes, and craniofacial deformities. Ultimately, 119 images (33 males and 86 females) met the criteria and were analyzed in the study.

Based on the ANB angles, the samples were categorized into three groups: Class I (*n* = 49), Class II (*n* = 52), and Class III (*n* = 18). The classifications were defined as follows: ANB < 0 for Class III, 0 < ANB < 4 for Class I, and ANB > 4 for Class II. For borderline cases, an additional Wits analysis was performed. In this analysis, Points A and B were projected perpendicularly onto the functional occlusal plane in the midsagittal section of the three-dimensional view, and the distance between these points was measured. Individuals were categorized as Class I if the measurement ranged from −1 to 1, Class II if greater than 1, and Class III if less than −1. In cases where discrepancies existed between Steiner and Wits analyses, the Wits analysis was used as the primary reference.

**Figure 1:**
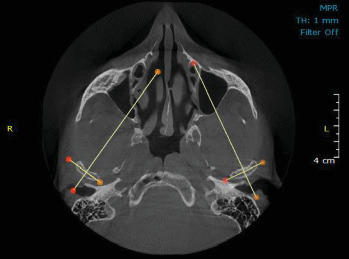
The horizontal reference axis of the condyle.

The CBCT images were acquired using a CBCT device (HDX-WILL, South Korea) with a field of view (FOV) of 14.5 cm × 16 cm and exposure settings of 110 kVp, 17 s exposure time, voxel size of 300 µm, and a current of 4 mA. Participants were instructed to remain still, refrain from swallowing, and maintain centric occlusion with relaxed lips and tongue during imaging. The images were analyzed using the OnDemand3D software (version 10.1). The Frankfurt plane was aligned parallel to the horizontal plane. The horizontal reference axis of the condyle was established by selecting the innermost and outermost points of the condyle head in the axial section (Figure 1). Based on this reference, the condyle and articular eminence were examined in three planes: axial, coronal, and sagittal, for both the right and left joints.

**Figure 2:**
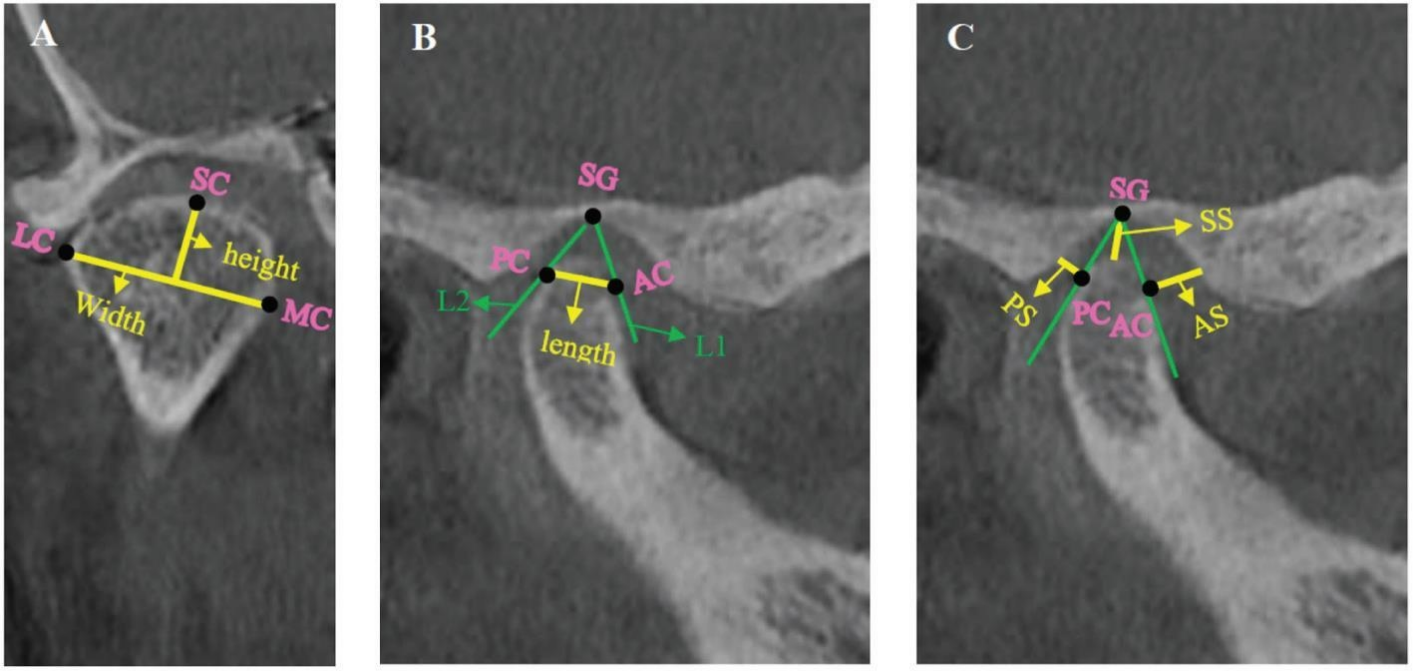
Landmarks and measurements. Condylar width and height (A): LC—the most lateral point of the condylar head; SC—the highest point of the condylar head; MC—the most medial point of the condylar head. Condylar length (B) AC—the anterior edge of the condylar head; SG—the top of the glenoid fossa; PC—the posterior edge of the condylar head. Superior (SS), anterior (AS), and posterior (PS) articular spaces (C).

In the sagittal plane, the upper joint space was determined by measuring the distance between the SG point (located at the top of the glenoid fossa) and the SC point (at the highest point of the condyle head; Figure 2). Additionally, in this plane, a line tangent to the anterior edge of the condyle head was drawn from the SG point, intersecting at a point designated as AC. A perpendicular line from AC to the anterior wall of the glenoid fossa was measured as the anterior articular space (Figure 2). Similarly, a tangent line from the SG point to the posterior limit of the condyle head identified a point called PC. A perpendicular line from PC to the posterior wall of the glenoid fossa was measured as the posterior articular space [22] (Figure 2).

In the coronal plane, from the SC point, a line was drawn perpendicular to a line connecting the MC point (the most medial part of the condyle head) and the LC point (the most lateral part of the condyle head), measuring the height of the condyle (Figure 2). The width of the condyle was determined by measuring the distance between the MC and LC points in the coronal plane (Figure 2). In the sagittal plane, the length of the condyle was measured as the distance between the AC and PC points [1] (Figure 2).

### 2.1. Sample Size Calculation

The sample size for this study was determined using PASS15 software for a one-way ANOVA design. The parameters used for calculation were a type I error rate (*α*) of 0.05 and a type II error rate (*β*) of 0.2 (80% power). The following parameters were considered for the main dependent variable:

• Mean for Group 1 (*μ*_1_): 18.48
• Mean for Group 2 (*μ*_2_): 17.01
• Mean for Group 3 (*μ*_3_): 18.55
• Standard deviation (*σ*): 2.78

Based on these parameters, the sample size calculation for each group was conducted using the formula for a one-way ANOVA:

  $$\begin{array}{c} n=\frac{2\sigma^{2}{\left(Z_{\alpha /3}+Z_{\beta }\right)}^{2}}{{\left(\mu_{1}-\mu_{2}\right)}^{2}}, \end{array}$$

where *Z*_*α*/3_ is the critical value of the standard normal distribution at *α*/3 to adjust for multiple comparisons (for three groups).

Using the provided parameters, the calculated sample size per group was 51. Accounting for potential dropouts and to ensure adequate power, a total sample size of 153 participants (51 per group) was determined.

### 2.2. Statistical Analysis

The position (superior–anterior–posterior articular space) and size (width–height–length of the condyle) were measured for each individual on both the right and left sides using OnDemand software. The average measurements were recorded. Statistical analyses were performed using SPSS software (version 24.0 for Windows). The Shapiro–Wilk test was utilized to assess the normality of data distribution, while Levene's test was applied to evaluate the equality of variances. A *T*-test was conducted to compare gender differences across various skeletal patterns. The Bonferroni method was employed for pairwise comparisons of the dependent variables across different skeletal patterns. An analysis of covariance (ANCOVA) was used to examine the effects of variables such as gender and different skeletal patterns on the dependent variables, adjusting for the impact of age. Statistical significance was set at *p*  < 0.05.

## 3. Result

CBCT images of 119 patients, consisting of 86 women (72.5%) and 33 men (27.5%), were analyzed. The participants ranged in age from 16 to 77 years, with an average age of 32.86 years (Table 1).

Different skeletal patterns did not show a statistically significant effect on the superior space (SS; *p* = 0.392). However, a statistically significant difference in SS was observed between genders, with men exhibiting a higher SS compared to women (*p* ≤ 0.001). The interaction between gender and skeletal patterns was not statistically significant (*p* = 0.337), but SS values were consistently higher in men than in women. The analysis indicated that the impact of different skeletal patterns was not significant in women (*p* = 0.443) or men (*p* = 0.367; Table 2).

Similarly, different skeletal patterns (*p* = 0.481), gender (*p* = 0.386), and the interaction between them (*p* = 0.257) did not significantly affect the anterior space (AS). The influence of different skeletal patterns was not significant in women (*p* = 0.236) or men (*p* = 0.196; Table 2). The evaluation of gender effects within each skeletal pattern group revealed a statistically significant difference only in the Class I group (Table 3).

For posterior space (PS), different skeletal patterns (*p* = 0.027) and gender (*p* = 0.001) had statistically significant effects. However, the interaction between them did not significantly impact PS (*p* = 0.556). PS values were consistently higher in men than in women, with the skeletal pattern differences ranked as follows: Class III > Class II > Class I. A significant difference was noted only between the Class I and Class III groups, with the PS value being lower in Class I compared to Class III; no other group comparisons showed statistically significant differences (Table 4).

There were no significant effects of different skeletal patterns (*p* = 0.367), gender (*p* = 0.100), or the interaction between them (*p* = 0.825) on the height of the condyle (Table 5). However, gender had a significant effect on condyle width, with men consistently showing a larger condyle width than women (*p* ≤ 0.001). The interaction between gender and skeletal patterns did not significantly affect condyle width (*p* = 0.720; Table 5). Different skeletal patterns significantly influenced condyle length (*p* = 0.002), though gender (*p* = 0.198) and the interaction between gender and skeletal patterns (*p* = 0.851) did not (Table 5). Significant differences in condyle length were observed between Class I and Class II and between Class II and Class III, with Class II exhibiting the lowest values (Table 6).

## 4. Discussion

Our study investigated the relationship between the morphology and position of the condyle and different skeletal patterns in the sagittal dimension using CBCT images. The morphology and position of the condyle are critical features in diagnosing and planning treatment for issues related to the TMJ. Various factors, such as gender, erosion, hyperplasia, and different syndromes, can affect the morphology of the condyle. The impact of condylar morphology and position on the function of the masticatory system and the development of malocclusions has been extensively researched. However, comprehensive knowledge of the relationship between TMJ morphology and various types of malocclusions, especially in the Iranian population, remains limited.

Systemic diseases, such as RA, can significantly alter the dimensions and positioning of TMJ structures. Youssef Mohamed et al. [32] identified that patients with RA exhibited significantly reduced condylar height and increased radiographic osteoarthritic changes compared to a control group. Additionally, there was a significant inverse correlation between anticitrullinated protein (ACCP) levels, disease activity score 28 (DAS28), and high to moderate disease activity with condylar anteroposterior dimensions. Consistent with these findings, our study demonstrated that individuals with a Class II skeletal pattern had significantly shorter condylar lengths compared to other skeletal groups. Moreover, our results corroborated Youssef Mohamed et al.'s [32] observation that ACCP levels did not significantly correlate with mediolateral dimensions. Furthermore, we found no statistically significant differences in condylar height and width across various skeletal patterns.

Previous research has provided important insights into how TMJ morphology is clinically relevant in individuals with different skeletal malocclusions. Variations in condylar position and joint space across skeletal classes may contribute to an increased risk of TMDs in specific populations. For instance, Song et al. [1] found that Class III malocclusions were linked to more anteriorly positioned condyles and reduced joint space, which could lead to higher stress on the TMJ and worsen TMD symptoms. In contrast, Alhammadi, Fayed, and Labib [11] found that Class II patients exhibited larger condylar height and anterior joint space, potentially presenting different biomechanical challenges. Additionally, they highlighted the importance of understanding the three-dimensional aspects of TMJ morphology, as both Class II and Class III patients showed notable differences in condylar and fossa dimensions, which may influence orthodontic treatment decisions and TMD management.

In alignment with Chae et al. [22], the current study found no statistically significant differences between the size of the upper joint space and different skeletal patterns. Akbulut and Kılınç [33] reported that the superior joint space, defined as the distance from the condyle to the fossa, had the highest values in Class II subjects on both sides, with statistical significance on the right side but not on the left. Tariq and Jan [34] observed that the upper joint space was smallest in Class III subjects and largest in Class I subjects. Arieta-Miranda et al. [35] and Song et al. [1] also reported the highest values in Class I individuals, but contrary to their findings, did not find significant differences between Class II and Class III individuals. In our study, there was no statistically significant difference between the size of the anterior joint space and malocclusion, consistent with studies conducted by Chae et al. [22] and Song et al. [1]. Arieta-Miranda et al. [35] used different landmarks from our study to measure the anterior space and concluded that Class I individuals have the largest amount of anterior space compared to Class II and Class III individuals. Posterior joint space values in Class I individuals were significantly lower than those in Class III subjects, aligning with the findings of Tariq and Jan [34]. Contrary to our results, studies by Chae et al. [22], Akbulut and Kılınç [33], and Arieta-Miranda et al. [35] found no statistically significant differences in the size of the posterior joint space across different skeletal patterns. Akbulut and Kılınç [33] and Arieta-Miranda et al. [35] used the external auditory canal as the posterior border of the posterior joint space. Discrepancies in joint space size between our study and others can be attributed to differences in sample size and racial variations.

This study found no statistically significant differences in condylar height across different skeletal patterns. In contrast, Song et al. [1] reported that Class I individuals had the highest condylar height values, using the same measurement method as ours. This difference may be attributed to racial variations. Hasebe et al. [7] and Santander et al. [2], who used different landmarks than the present study to define condylar height, concluded that condylar height was greatest in Class III individuals. There was no statistically significant difference in condylar width across different skeletal patterns, similar to the findings of Chae et al. [22]. However, other studies have reported different results. Our study found a statistically significant relationship between condylar length and different skeletal patterns, with Class II individuals exhibiting lower condylar length values than Class III and Class I individuals. Hasebe et al. [7] and Tariq and Jan [34] measured condylar length using the AC and PC points, selected on the anterior and posterior limits of the condyle head at a distance of 4 mm from the uppermost point of the condyle head in the sagittal dimension, and found no statistically significant difference between the three groups. In contrast, Song et al. [1], using the most anterior and posterior points of the condyle head in the axial dimension, concluded that Class I individuals have the highest values. Santander et al. [2] and Chae et al. [22] used methods similar to ours, connecting the most anterior and posterior points of the condyle head in the sagittal dimension. Santander et al. [2] concluded that Class III individuals have the lowest values, whereas Chae et al. [22] found no statistically significant differences between the three groups. The differences in results between our study and others may be due to variations in measurement methods and inclusion and exclusion criteria. For example, Chae et al. [22] excluded cases with discrepancies between CO-CR and underlying diseases affecting metabolism, which were not considered in the present study.

A limitation of this study is the unequal distribution of male and female participants across skeletal classes, with an overrepresentation of Class II in females. This imbalance may affect the generalizability of the findings and limiting their interpretability. Future studies with a more balanced gender distribution are recommended for more reliable conclusions on the relationship between TMJ morphology and skeletal patterns. The limited number of Class III individuals and the lack of access to patient histories to account for other potential factors, such as TMD, in the development of skeletal malocclusions were another limitations of this study. Future studies should include larger sample sizes from various regions and consider additional factors that may influence the development of different skeletal patterns in the sagittal, vertical, and transverse dimensions.

## 5. Conclusion

Based on the results of this study, Class I individuals had smaller posterior joint space compared to Class III individuals, while Class II individuals had shorter condyle length than both Class I and Class III. These findings emphasize the role of skeletal patterns in TMJ morphology, with potential implications for orthodontic treatment and TMJ disorder management.

## Figures and Tables

**Table 1:** Statistical indices of age in the three skeletal pattern groups.

Skeletal pattern	Mean	*N*	Standard deviation
Class I	32.90	49	9.514
Class II	34.58	52	11.699
Class III	27.78	18	8.264
Total	32.86	119	10.538

**Table 2:** Comparing statistical indices for the superior, anterior, and posterior joint spaces in various skeletal patterns by gender.

Sex	Skeletal pattern	Superior joint space	Anterior joint space	Posterior joint space	*N*
F	Class I	Mean: 3.0467 SD (0.92148)	Mean: 2.3275 SD (0.76095)	Mean: 1.8239 SD (0.56306)	38
	Class II	Mean: 3.1484 SD (0.96333)	Mean: 2.6850 SD (1.05093)	Mean: 1.8963 SD (0.50087)	41
	Class III	Mean: 3.4157 SD (0.99305)	Mean: 2.5871 SD (1.02596)	Mean: 2.3386 SD (0.87262)	7
	Total	Mean: 3.1252 SD (0.94123)	Mean: 2.5191 SD (0.93604)	Mean: 1.9003 SD (0.57293)	86

M	Class I	Mean: 3.8355 SD (0.65824)	Mean: 2.9591 SD (0.86686)	Mean: 2.2255 SD (0.70146)	11
	Class II	Mean: 4.4318 SD (0.60191)	Mean: 2.8932 SD (1.17736)	Mean: 2.7932 SD (1.06419)	11
	Class III	Mean: 3.8177 SD (1.09025)	Mean: 2.3055 SD (0.74753)	Mean: 2.7336 SD (1.03037)	11
	Total	Mean: 4.0283 SD (0.83909)	Mean: 2.7192 SD (0.96523)	Mean: 2.5841 SD (0.95203)	33

Total	Class I	Mean: 3.2238 SD (0.92486)	Mean: 2.4693 SD (0.82085)	Mean: 1.9141 SD (0.61282)	49
	Class II	Mean: 3.4199 SD (1.03875)	Mean: 2.7290 SD (1.07024)	Mean: 2.0861 SD (0.74539)	52
	Class III	Mean: 3.6614 SD (1.04304)	Mean: 2.4150 SD (0.84863)	Mean: 2.5800 SD (0.96568)	18
	Total	Mean: 3.3757 SD (0.99696)	Mean: 2.5746 SD (0.94440)	Mean: 2.0900 SD (0.75943)	119

Abbreviations: F, female; M, male; SD, standard deviation.

**Table 3:** *T*-test to evaluate the effect of gender in different skeletal patterns.

Skeletal pattern	*T*-test	*p*-Value
Class I	2.426	0.019
Class II	0.569	0.572
Class III	−0.675	0.509

**Table 4:** Bonferroni test to compare two by two posterior space (PS) in different skeletal patterns.

(I) Skeletal pattern	(J) Skeletal pattern	Mean difference (I−J)	Standard error	*p*-Value
Class I	Class II	−0.265	0.164	0.330
	Class III	−0.578	0.202	0.015

Class II	Class III	−0.313	0.207	0.396

**Table 5:** Comparing statistical indices for the height, width, and length of condyle in various skeletal patterns by gender.

Sex	Skeletal pattern	Height	Width	Length	*N*
Female	Class I	Mean: 5.4861 SD (0.00135)	Mean: 18.4704 SD (1.84261)	Mean: 6.2549 SD (1.08728)	38
	Class II	Mean: 5.2367 SD (0.86093)	Mean: 2.34844 SD (1.05093)	Mean: 5.6952 SD (0.80428)	41
	Class III	Mean: 5.3793 SD (0.72871)	Mean: 18.8721 SD (1.46342)	Mean: 6.4000 SD (1.06498)	7
	Total	Mean: 5.3585 SD (0.91500)	Mean: 18.6781 SD (2.06376)	Mean: 5.9999 SD (0.99301)	86

Male	Class I	Mean: 5.7314 SD (1.12871)	Mean: 21.1755 SD (3.10130)	Mean: 6.4609 SD (1.22181)	11
	Class II	Mean: 5.5723 SD (0.84237)	Mean: 21.2464 SD (2.14809)	Mean: 5.9936 SD (1.20063)	11
	Class III	Mean: 5.9750 SD (1.26319)	Mean: 18.8721 SD (1.46342)	Mean: 6.9059 SD (0.80967)	11
	Total	Mean: 5.7595 SD (1.07089)	Mean: 21.5132 SD (2.58029)	Mean: 6.4535 SD (1.12469)	33

Total	Class I	Mean: 3.2238 SD (0.92486)	Mean: 19.0777 SD (2.43338)	Mean: 6.3011 SD (1.10897)	49
	Class II	Mean: 3.4199 SD (1.03875)	Mean: 19.3470 SD (2.49343)	Mean: 5.7584 SD (0.89730)	52
	Class III	Mean: 3.6614 SD (1.04304)	Mean: 20.8556 SD (2.68527)	Mean: 6.7092 SD (0.92213)	18
	Total	Mean: 5.4697 SD (0.97293)	Mean: 19.4643 SD (2.54912)	Mean: 6.1257 SD (1.04639)	119

Abbreviation: SD, standard deviation.

**Table 6:** Bonferroni test to compare two by two length of the condyle in different skeletal patterns.

(I) Skeletal pattern	(J) Skeletal pattern	Mean difference (I−J)	Standard error	*p*-Value
Class I	Class II	−0.601	0.238	0.039
	Class III	−0.401	0.292	0.518

Class II	Class III	−1.002	0.299	0.003

## Data Availability

The data supporting the findings of this study are available from the corresponding author upon reasonable request.
